# Ligand Discovery for the Alanine-Serine-Cysteine Transporter (ASCT2, SLC1A5) from Homology Modeling and Virtual Screening

**DOI:** 10.1371/journal.pcbi.1004477

**Published:** 2015-10-07

**Authors:** Claire Colas, Christof Grewer, Nicholas James Otte, Armanda Gameiro, Thomas Albers, Kurnvir Singh, Helen Shere, Massimiliano Bonomi, Jeff Holst, Avner Schlessinger

**Affiliations:** 1 Department of Pharmacology and Systems Therapeutics, Tisch Cancer Institute, Icahn School of Medicine at Mount Sinai, New York, New York, United States of America; 2 Department of Chemistry, Binghamton University, Binghamton, New York, United States of America; 3 Origins of Cancer Laboratory Centenary Program, Camperdown, Australia; 4 Sydney Medical School, University of Sydney, Sydney, Australia; 5 Department of Chemistry, University of Cambridge, Cambridge, United Kingdom; Tel Aviv University, ISRAEL

## Abstract

The Alanine-Serine-Cysteine transporter ASCT2 (SLC1A5) is a membrane protein that transports neutral amino acids into cells in exchange for outward movement of intracellular amino acids. ASCT2 is highly expressed in peripheral tissues such as the lung and intestines where it contributes to the homeostasis of intracellular concentrations of neutral amino acids. ASCT2 also plays an important role in the development of a variety of cancers such as melanoma by transporting amino acid nutrients such as glutamine into the proliferating tumors. Therefore, ASCT2 is a key drug target with potentially great pharmacological importance. Here, we identify seven ASCT2 ligands by computational modeling and experimental testing. In particular, we construct homology models based on crystallographic structures of the aspartate transporter Glt_Ph_ in two different conformations. Optimization of the models’ binding sites for protein-ligand complementarity reveals new putative pockets that can be targeted via structure-based drug design. Virtual screening of drugs, metabolites, fragments-like, and lead-like molecules from the ZINC database, followed by experimental testing of 14 top hits with functional measurements using electrophysiological methods reveals seven ligands, including five activators and two inhibitors. For example, aminooxetane-3-carboxylate is a more efficient activator than any other known ASCT2 natural or unnatural substrate. Furthermore, two of the hits inhibited ASCT2 mediated glutamine uptake and proliferation of a melanoma cancer cell line. Our results improve our understanding of how substrate specificity is determined in amino acid transporters, as well as provide novel scaffolds for developing chemical tools targeting ASCT2, an emerging therapeutic target for cancer and neurological disorders.

## Author Summary

ASCT2 is a membrane protein that imports neutral amino acids into cells in exchange for intracellular amino acids. ASCT2 is highly expressed in peripheral tissues such as the lung, where it contributes to the homeostasis of intracellular concentrations of neutral amino acids. Recently, ASCT2 has been shown to be important for nutrient uptake in reprogrammed cancer networks. Here, we use an innovative computational approach that includes homology modeling and ligand docking to model the structure of this transporter in two distinct conformations, and virtually screen large compound libraries against these models. We use a variety of experimental assays and functional measurements to confirm seven new ligands for this transporter, including five activators and two inhibitors. This combined approach reveals specificity determinants for ligand-binding and transport, including previously unknown pockets to be targeted via structure-based drug design. The results improve our understanding of how substrate specificity is determined in amino acid transporters and provide a framework for developing potent chemical tools and potential drugs targeting ASCT2, an emerging therapeutic target for cancer and neurological disorders.

## Introduction

The solute carrier 1 family (SLC1) consists of five glutamate transporters (Excitatory Amino Acid Transporters, EAATs) that contribute to the regulation of synaptic concentrations of glutamate—the primary excitatory neurotransmitter in the central nervous system (CNS); and two neutral amino acid transporters (Alanine-Serine-Cysteine transporters, ASCT1 and 2) that exchange amino acids in neurons and/or cells of the peripheral tissues, to contribute to the homeostasis of intracellular concentrations of neutral amino acids [1]. ASCT2 (SLC1A5) is a sodium-dependent transporter located in the lung, kidney, intestines, and testis, where it transports small neutral amino acids across the cell membrane. ASCT2 expression levels are increased in various types of cancer, including glioblastoma multiforme (GBM) [2], neuroblastoma [3], lung cancer [4], prostate cancer [5] and melanoma [6]. ASCT2 was suggested to play a key role in cancer metabolism by supplying growing tumor cells with amino acids that are used as nutrients to build biomass and as signaling molecules to activate growth and proliferation pathways such as the mTOR pathway [7,8]. Thus, ASCT2 is a potential cancer drug target, where a compound interacting with ASCT2 can be an inhibitor that deprives the cancer cells of nutrients, a cytotoxic ASCT2 substrate with an intracellular target (e.g., a metabolic enzyme), or a low affinity ligand (a substrate or inhibitor) that acts as inhibitor or substrate on multiple targets, including ASCT2 [9].

Currently, no experimentally determined atomic structures for any of the human SLC1 family members, including ASCT2, are known. However, structures of an SLC1 homolog, the aspartate transporter Glt_Ph_, from the archaean organism *Pyrococcus horikoshii*, have been determined in different conformations of the transport cycle [10,11]. Glt_Ph_ shares 24–35% sequence identity and the same number of transmembrane helices (i.e., eight) with the human SLC1 family, as well as a conserved binding site; therefore the Glt_Ph_ structure is the most suitable template for generating homology models of the SLC1 members [1,10,12]. Indeed, previous homology models for various human SLC1 family members [13–15] have revealed important structure-function relationships in the SLC1 family. For example, Scopelliti *et al*. have recently converted the substrate specificity of ASCT1 from transporting neutral amino acids to transporting glutamate by mutating three binding site amino acids (e.g., A382T), revealing previously unknown specificity determinants for the SLC1 family [15].

In addition, structure determination of Glt_Ph_ structures in different conformations with X-ray crystallography [10,11] and experimental characterization of this protein using other approaches (e.g., double electron-electron resonance spectroscopy [16]) have contributed to our understanding of the dynamics of the SLC1 family. For example, it was shown that Glt_Ph_ exists in a conformational ensemble of protomers that sample the outward-facing and inward-facing states with nearly equal probabilities, and that specific mutants adopt unique conformations [16]. These studies, together with computational analyses [17,18], confirm that Glt_Ph_, and likely the human SLC1 family members, including ASCT2, transport ligands across the cell membrane *via* the ‘alternating access’ transport mechanism in which the transporter undergoes conformational changes between extracellular outward-facing and intracellular inward-facing states, and the substrate binding sites can be exposed to either side of the membrane [19]. Particularly, it has been suggested that Glt_Ph_ transports substrates via an “elevator mechanism”, where one domain remains static while the transport domain makes substantial movement from the extracellular side to the intracellular side as a rigid-body [20]. Two hairpin loops, HP1 on the intracellular side and HP2 on the extracellular side, act as gates that allow the release and binding of the substrate. Describing the structural basis for substrate specificity in the SLC1 family and further characterization of this pharmacologically important transporter family is expected to expand our understanding of transport in human systems as well as help in the design of drugs for metabolic diseases and cancer.

Here, we characterize ASCT2 using a combined computational and experimental approach [21–24]. We construct structural models of ASCT2 based on structures of Glt_Ph_ in two different conformations, and refine the models to distinguish between known ligands and likely non-binders. We perform virtual ligand screening of various small molecule libraries against these models, where top scoring hits are tested experimentally for ASCT2 inhibition and activation using electrophysiological methods, as well as for their effect on melanoma cell line proliferation and apoptosis. Finally, we describe how the results of this study improve our understanding of the chemical basis for discriminating inhibitors from activators for ASCT2, as well as discuss the pharmacological implications of our results, including the potential use of the identified ligands as chemical tools to characterize the role of ASCT2 in cancer.

## Results and Discussion

### ASCT2 homology models

**Fig 1:**
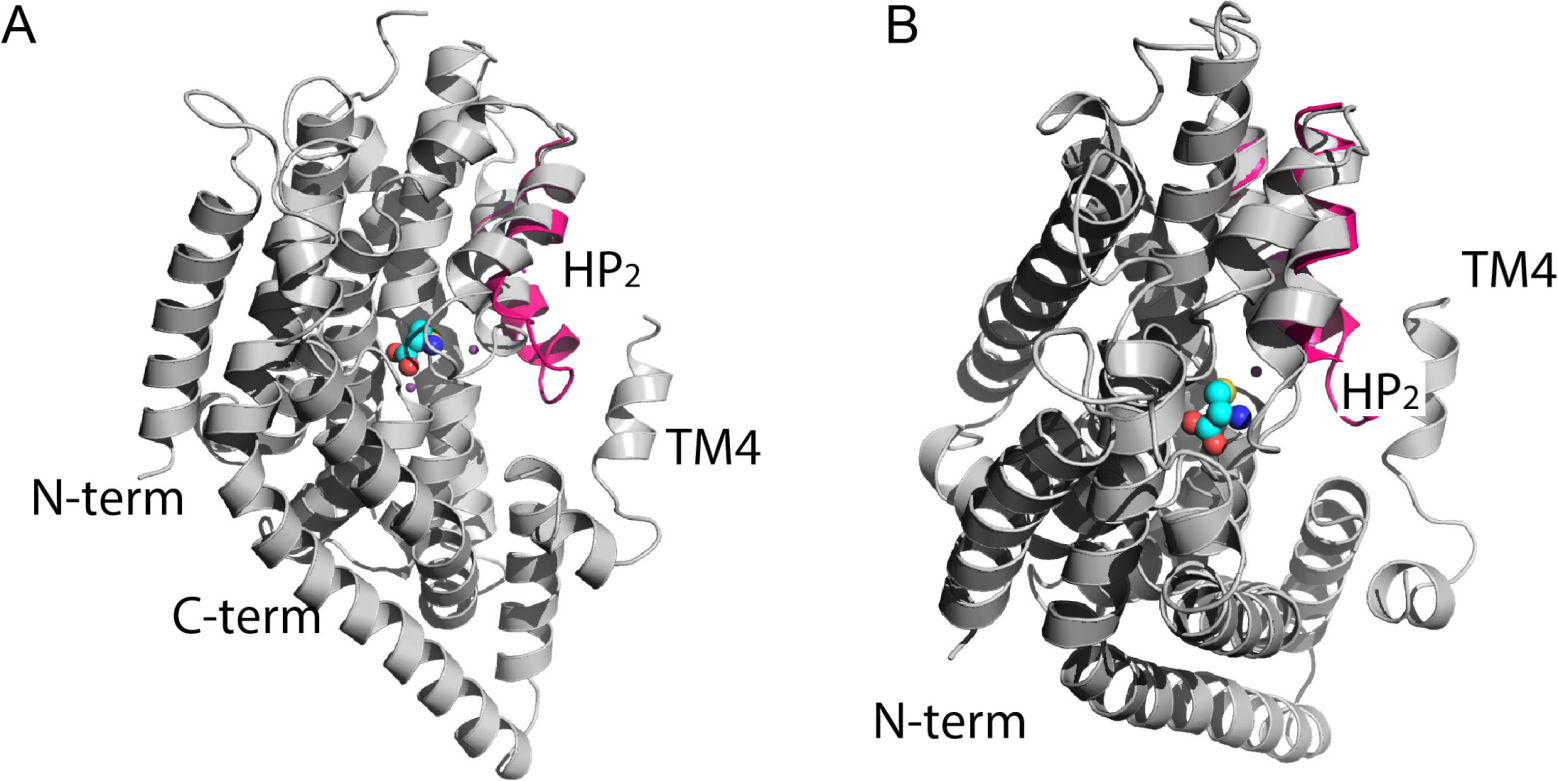
ASCT2 models in different conformations reveal gating mechanism of the HP2 loop. Side (**A**) and cytoplasmic (**B**) view of the ASCT2 models in the occluded conformation are represented in gray ribbons. The HP2 loop of the outward-open conformation (pink ribbons) is superposed to the occluded model. Atoms of the substrate cysteine are shown as spheres where oxygen atoms are displayed in red, sulfur in yellow, and carbon atoms in cyan. Sodium ions are illustrated as small purple spheres.

We modeled the ASCT2 structure using MODELLER [25] based on the Glt_Ph_ structures (sequence identity of ~24%) in two different conformations, including an outward-occluded (‘occluded’) state and an inhibitor-bound outward open (‘outward-open’) state [10,11] (Methods). The ASCT2 models contain eight transmembrane helices that make up the entire transmembrane region of the protein, as well as one ligand and two sodium ions, which their initial coordinates were derived from their location on the template structures (Fig 1). Next, the ASCT2 models in each state were optimized for protein-ligand complementarity by iteratively sampling different conformations from the initial MODELLER’s models, refining these models by sidechain modeling on a fixed backbone with SCWRL4 [26], performing minimization with molecular dynamics (MD) simulations with GROMACS [27], and evaluating how well the models can discriminate known ligands from decoys with enrichment calculations (Methods).

**Fig 2:**
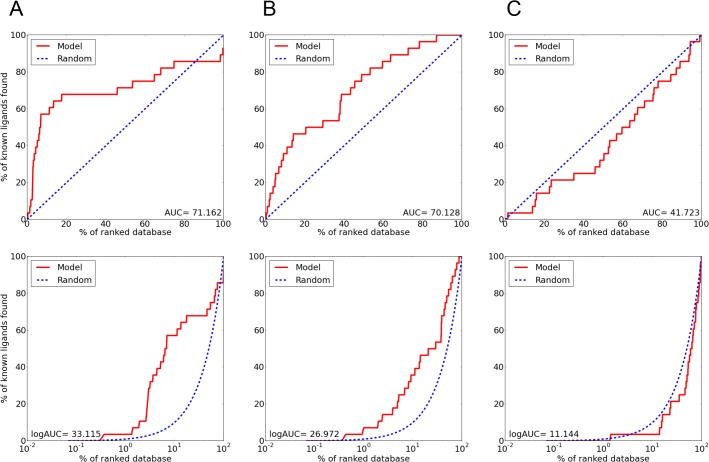
Enrichment plots of the ASCT2 models indicate the suitability of the models for productive virtual screening. Enrichment plots of ASCT2 in **(A)** the occluded conformation, **(B)** the outward open conformation and **(C)** the outward open conformation with Asp460 facing the binding site. The enrichment plots are represented in red, whereas the plot that is expected by random selection of ligands is represented in a dashed blue line. The bottom panel shows the enrichment in a semi-logarithmic scale.

The final models obtained logAUC values of 33.1 (occluded conformation) and 27 (outward-open conformation) (Fig 2), suggesting that both conformations can be used for productive virtual screening and that the occluded ASCT2 model captures interactions with ligands more accurately than the outward-open model [23,28]. These final models include two helical hairpin loops (referred to as HP1 and HP2), which are inserted into the membrane from its opposing sides, similar to the equivalent loops in the Glt_Ph_ structures. In the occluded state, HP2 occludes the substrate from the aqueous environment, while in the outward-open state HP2 adopts an open conformation and may act as the extracellular gate (Fig 1). Thus, the outward-open state has a significantly larger surface area in the ligand binding site despite overall high structural similarity (RMSD of 0.5 Å).

### Occluded state predicts non-trivial substrate-like compounds

**Fig 3:**
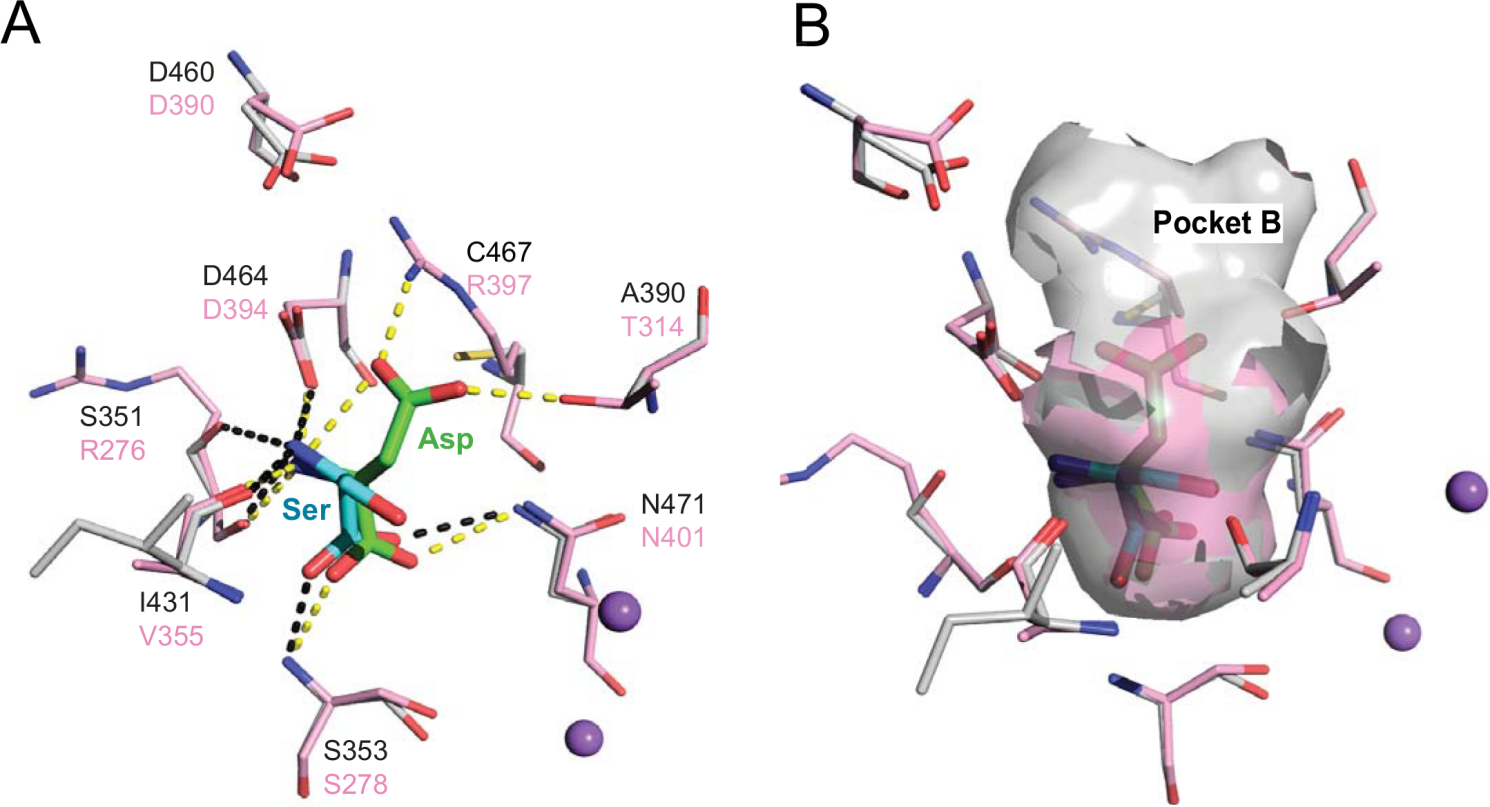
Binding sites of ASCT2 and Glt_Ph_ differ in their shape, size, and polarity. The final model of ASCT2 (gray) in an occluded conformation is superimposed on the X-ray structure of Glt_Ph_ (pink). Key residues are displayed as lines, where oxygen and nitrogen atoms are colored in red and blue, respectively; the sodium ions are visualized as purple spheres. The L-aspartate coordinates from the Glt_Ph_ structure are depicted by green sticks and L-serine coordinates derived from the docking of known ligands against the ASCT2 model are visualized with cyan sticks. (**A**) Hydrogen bonds between L-aspartate and Glt_Ph_ are shown in dotted yellow lines and between L-serine and the ASCT2 model in dotted black lines. (**B**) The surface of the binding pocket is displayed in pink and gray for the template and the model, respectively, to visualize the additional pocket (pocket B) accessible in the model compared to the Glt_Ph_ structure.

**Fig 4:**
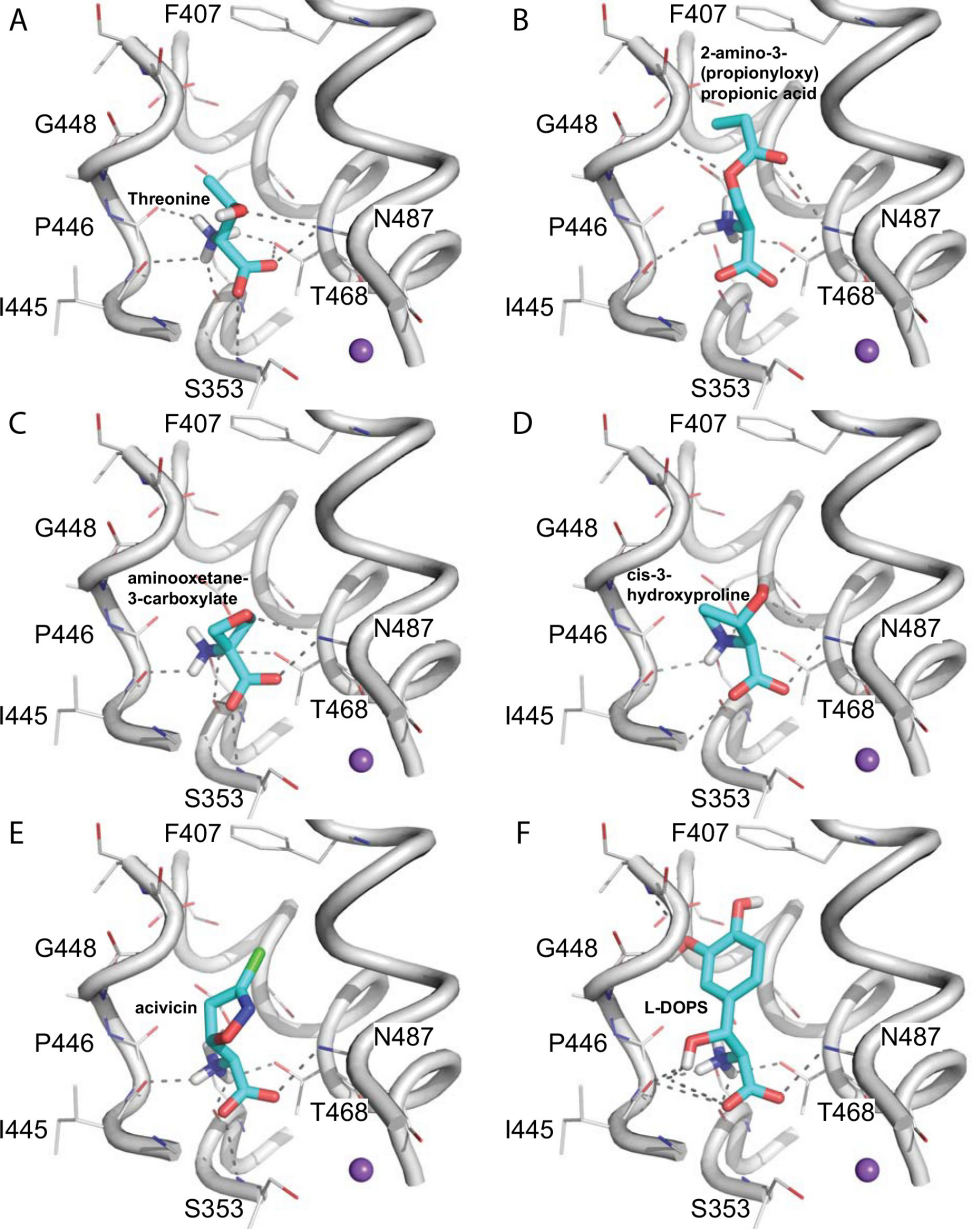
Ligand binding mode for the ASCT2 occluded conformation model reveals key residues for substrate binding. Predicted binding modes of selected known ligands (**A-B**) and ligands identified in this study (**C-F**). The backbone atoms of the ASCT2 binding site are visualized in gray cartoon; sidechain atoms of key residues are illustrated with gray lines and ligands are displayed as cyan sticks, with oxygen, nitrogen, and hydrogen atoms in red, blue, and white, respectively; hydrogen bonds between the small molecules and ASCT2 (involving residues Phe407, Gly448, Pro446, Ile445, Ser353, Thr468, Asn471) are displayed as dotted gray lines. The small molecule ligands are Threonine (**A**), 2-amino-3-(propionyloxy)propionic acid (**B**), aminooxetane-3-carboxylate (**C**), *cis*-3-hydroxyproline (**D**), acivicin (**E**), and L-DOPS (**F**).

We analyzed the predicted docking poses of known ligands against the models of the two conformations, and compared them to the Glt_Ph_ structure in complex with the substrate aspartate (occluded state) and the competitive inhibitor TBOA (outward-open state). The occluded ASCT2 conformation model reveals a small binding site that limits the size of the ligands and their mode of interaction with the binding site residues (Figs 3 and 4). In particular, the majority of the interactions between the binding site residues and known ASCT2 ligands are similar to those in the Glt_Ph_-aspartate complex structure. For example, the amino and carboxy groups of ASCT2 ligands such as serine (Fig 3A) or threonine (Fig 4A) make polar interactions with Ser353, Pro432, Ile431, Asp464, Thr468, and Asn471 of ASCT2, similar to the interactions that the corresponding chemical groups of the Glt_Ph_ ligand aspartate make with the Glt_Ph_ binding site residues (Arg278, Pro356, Val355, Asp394, Thr398, Asn401) (Fig 3A). Notably, the amino acid substitution of Arg397 in Glt_Ph_ to Cys467 in ASCT2 contributes significantly to ligand binding specificity among these transporters. In particular, the Glt_Ph_ binding site residues Arg397 and Asp390 form a salt bridge, in which the basic sidechain atoms of Arg397 also form hydrogen bonds with the β-carboxy group of the ligand aspartate. In ASCT2, however, Cys467 substitutes Arg397 of Glt_Ph_, breaking the salt bridge and changing the size, shape, and overall charge distribution of the binding site. As a result, the region, named “pocket B” (Fig 3B), occupied by the sidechain of Arg397 in Glt_Ph_, becomes accessible for ligands in ASCT2, where ligands can interact with Asp460 (which corresponds to Asp390 in Glt_Ph_) as well as with other, hydrophobic residues including Phe407 and Val477. Interestingly, in ASCT1, Thr459, which corresponds to Cys467 in ASCT2, was shown to play a key role in determining substrate specificity, further supporting the role of this position in mediating interactions with substrates [15].

We then docked various libraries from the ZINC [29] and KEGG [30,31] databases against the occluded structural model. We hypothesized that virtual screening against the occluded conformation model, which is based on the template structure bound to its substrate aspartate, will capture substrate-like ASCT2 ligands that can be activators or competitive inhibitors. Moreover, the binding site of the occluded conformation is small and narrow; thus, the predicted compounds that interact with this conformation are more likely to be amino-acid analogs. Indeed, various previously-characterized amino acid substrates of ASCT2 ligands were ranked highly in our screen, increasing our confidence in the approach. For instance, cysteine and threonine were ranked #14 and #24, respectively, in the KEGG DRUG docking screen of 6,436 molecules. The top 500 docking poses of molecules from the various ligand screens were visually analyzed in the context of the predicted pose of the known ligands (Fig 4A and 4B) [32,33].

Our strategy was to select molecules based on their ability to maintain important conserved interactions with binding site residues that are needed for function, while also exploring additional interactions and pockets for each conformation, similarly to approaches taken in previous successful studies [9,21,23]. Particularly, we picked molecules that form polar interactions with the binding site residues that were also predicted to interact with the carboxy and amino groups of the known ligands (e.g., Ser351, Ser353, Ile431, Asp464 and Asn471). Therefore, because most compounds are expected to consist of amino acid-like scaffold, to increase the probability of identifying potential novel scaffolds and reduce the bias introduced by the visual analysis, (i) we selected ligands exploring the newly discovered pocket B, constituted by several hydrophobic (Phe407 and Val477) and polar residues (Asp460), which is expected to increase the potential ligands chemical space that can be explored; (ii) We also focused on ‘non-trivial’ amino acid analogs that would unlikely be tested without being highly ranked by virtual screening. For example, proline is not a ligand of ASCT2 and we tested the proline analog cis-3-hydroxyproline because it formed additional key interactions with ASCT2 binding site (e.g. Asn471, Fig 4D). Finally, eight compounds were selected for experimental testing based on the occluded model (Fig 4C–4F and Table 1 and S1 Fig).

**Table 1:** Experimentally confirmed ligands.

Name^a^	I_max_ ^b^	K_m_ ^c^ (mM)		Tc^d^
Alanine (control)	1	0.41 ± 0.02		
**Occluded conformation**				
*cis*-3-hydroxyproline	1.39 ± 0.1	0.019 ± 0.01		0.51
Aminooxetanecarboxylate (AOC)	1.17 ± 0.13	0.22 ± 0.02		0.55
Penicillamine	0.68 ± 0.08	1.37 ± 0.68		0.64
Chloroalanine	1.28 ± 0.22	0.90 ± 0.12		0.62
Acivicin	0.45 ± 0.03	5.1 ± 1.5		**0.46**
L-DOPS	-0.03 ± 0.01	1.95 ± 0.9		0.55
**Outward-open conformation**				
**γ**-FBP	-0.64 ± 0.13	0.087 ± 0.022		**0.47**

a Name is the generic name of the molecule. Chemically novel ligands are marked with bold font

b I_max_ marks the maximum currents relative to 1 mM alanine

c K_m_ is the K_m_ values for the active compounds

d Tc is the Tanimoto coefficient calculated relying on Daylight fingerprints. Tc values of < 0.5 suggest that the molecule is a chemically novel ASCT2 ligand

### Experimental testing confirms ASCT2 activators

We used transporter-mediated anion current as a measure of transport/inhibition activity [34,35] (Methods), because, due to its function as an electroneutral obligate exchanger, ASCT2 does not mediate steady-state transport current. We have previously shown that anion current is a measure of transport activity [36], due to the kinetic coupling of transport and anion flux, and this fact is also well established for the homologous glutamate transporters [37], which have a much more rigorously determined pharmacology [38]. Anion current is activated when the transporter visits certain states in the transport cycle, such as the fully Na^+^/substrate occupied state (substrate-induced anion conductance), or the Na^+^-bound state (leak conductance) [39]. Thus, transported substrates induce anion current and non-transported inhibitors block the leak anion current. This kinetic link between transport and anion current can be demonstrated using a prototypical competitive inhibitor, benzylserine, which is unable to elicit exchange-mediated transient transport currents in ASCT2-expressing cells, in contrast to the transported substrate, alanine (S2 Fig) [39]. These transient currents are directly caused by re-equilibration of the amino acid translocation equilibrium upon voltage jumps and are, thus, a direct measure of transport. Functional characterization by patch clamping is a powerful approach when combined with virtual screening that prioritizes molecules from large libraries, but precludes screening of large amounts of compounds due to its labor-intensive nature.

**Fig 5:**
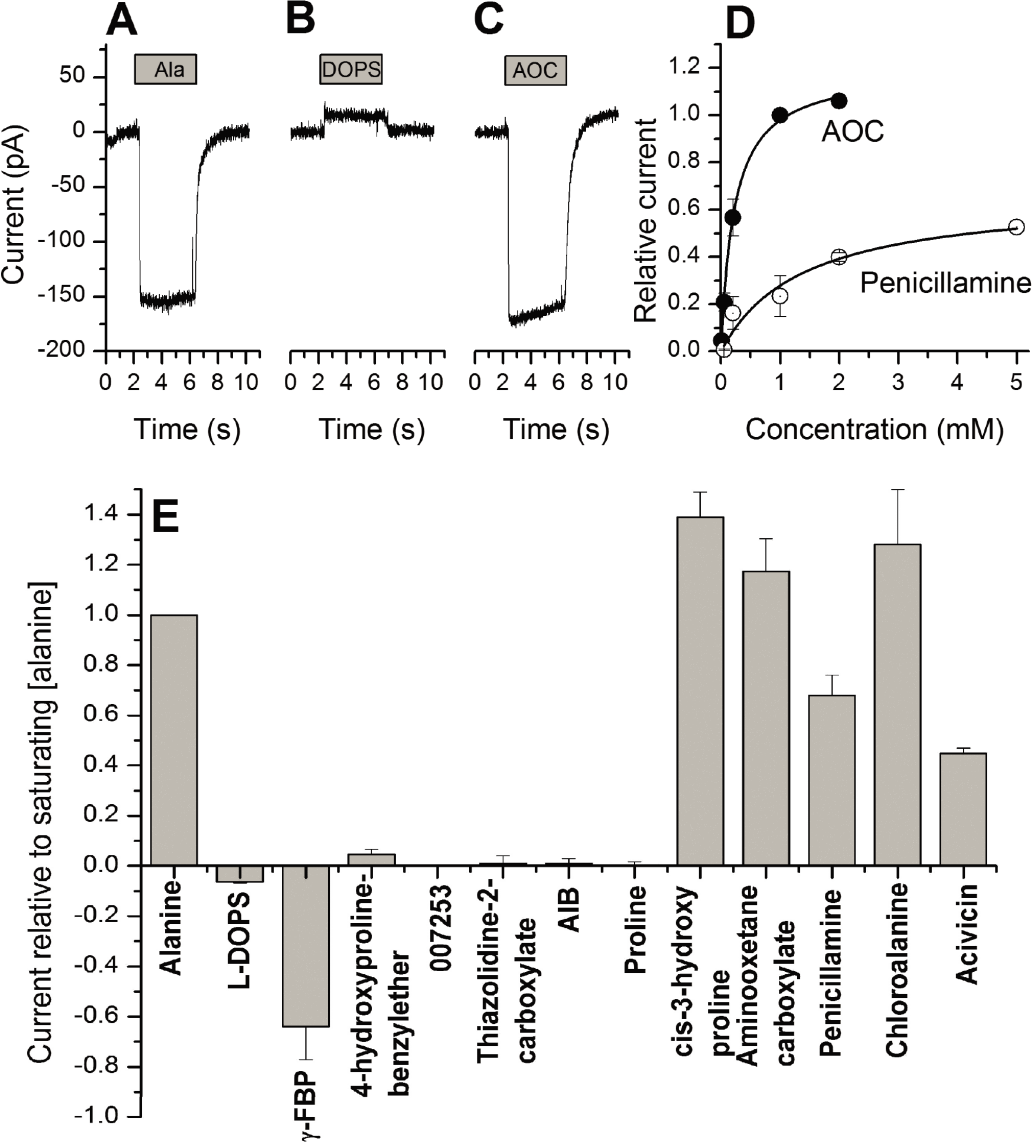
Electrophysiological methods confirm predicted activators and inhibitors. (**A**-**C**) Representative whole-cell current traces in response to 1 mM of alanine, L-DOPS, and aminooxetane-3-carboxylate (AOC) applied at the time indicated by the gray bar. (**D**) Dose response curves for AOC and penicillamine (membrane potential = 0 mV, internal buffer contained 130 mM NaSCN and 10 mM alanine, external buffer contained 140 mM NaCl). (**E**) Maximum whole-cell currents relative to that induced by 1 mM alanine (membrane potential = 0 mV, internal buffer contained 130 mM NaSCN and 10 mM alanine, external buffer contained 140 mM NaCl).

Inward anion currents were observed upon application for 5 of the 8 selected compounds, similarly to the native transported substrate alanine (Fig 5A and 5C and 5D), indicating that these compounds may be transported substrates (activators). One of these previously unknown ligands, the anti-cancer agent acivicin, is a chemically novel ASCT2 ligand (Tanimoto coefficient (Tc) of 0.46 to the most similar known ASCT2 ligand), although the apparent affinity is fairly low (Table 1). Two of the compounds (AIB and thiazolidine-2-carboxylate) did not elicit any significant response, at concentrations up to 5 mM, indicating that they do not interact with ASCT2. Two other compounds, N-(2-phenylethyl)tryptophan and 3-{[(1H-imidazol-2-ylmethyl)(methyl)amino]methyl}-5-methyl-1H-indole-2-carboxylic acid, were poorly soluble in water and required 5% DMSO for solubilization at concentrations higher than 1 mM. At the maximum concentration tested, 500 μM, these compounds were also inactive. They were not included in Fig 5E due to these solubility problems. Some other compounds, including chloroalanine, cis-3-hydroxyproline, and penicillamine activated inwardly-directed anion currents, three of them with a higher *I*_max_ than that of alanine (Table 1). These results indicate that these compounds are more effective activators of the ASCT2 anion conductance, and are, possibly, more rapidly transported. For example, *cis*-3-hydroxyproline induces a relative current of 1.39—the highest activity of all the active compounds (Fig 5E and Table 1). Surprisingly, *cis*-3-hydroxyproline is a proline derivative. Proline is a known non-ligand of ASCT2, and has been tested here as negative control (Fig 5E). The hydroxyl moiety of the proline establishes hydrogen bonds with residue Asn471, which likely contributes to the increased affinity of the compound (Fig 4D). These results demonstrate the strength of relatively unbiased structure-based virtual screening in capturing ligands that would unlikely be considered for testing otherwise. The overall structure of the newly identified ASCT2 activators includes a small neutral amino acid scaffold, in which an oxygen atom connected to the C_γ_ atom makes key polar interactions with binding site residues. This provides an initial pharmacophore model for designing better ASCT2 cytotoxic substrates for cancer therapy.

Finally, L-DOPS, was found to be a weak inhibitor of the ASCT2 leak anion conductance (Fig 5B and 5E), inducing apparent outward current by inhibiting leak anion outflow from the cell. L-DOPS current responses, although small, were always outward directed, indicating that the inhibitory effect, while small, is significant. In contrast, the currents elicited by 4-hydroxyproline-benzylether were too small to be measurable in most cells, or randomly inward or outward directed, suggesting that the response was not significantly different from zero. In addition of inducing only small outward current, L-DOPS also showed low apparent affinity, with a K_i_ of about 2 mM (Table 1). Due to this low affinity, L-DOPS was unable to significantly inhibit alanine-induced responses at concentrations up to 5 mM. The modulation of ASCT2 by acivicin and L-DOPS, both of which are also ligands of the L-type amino acid transporter 1 (LAT-1) [22], suggests that ASCT2 and LAT-1 have common ligands and that drugs targeting nutrient transporters can potentially be designed to interact with both proteins simultaneously.

### Outward facing model reveals new pocket

**Fig 6:**
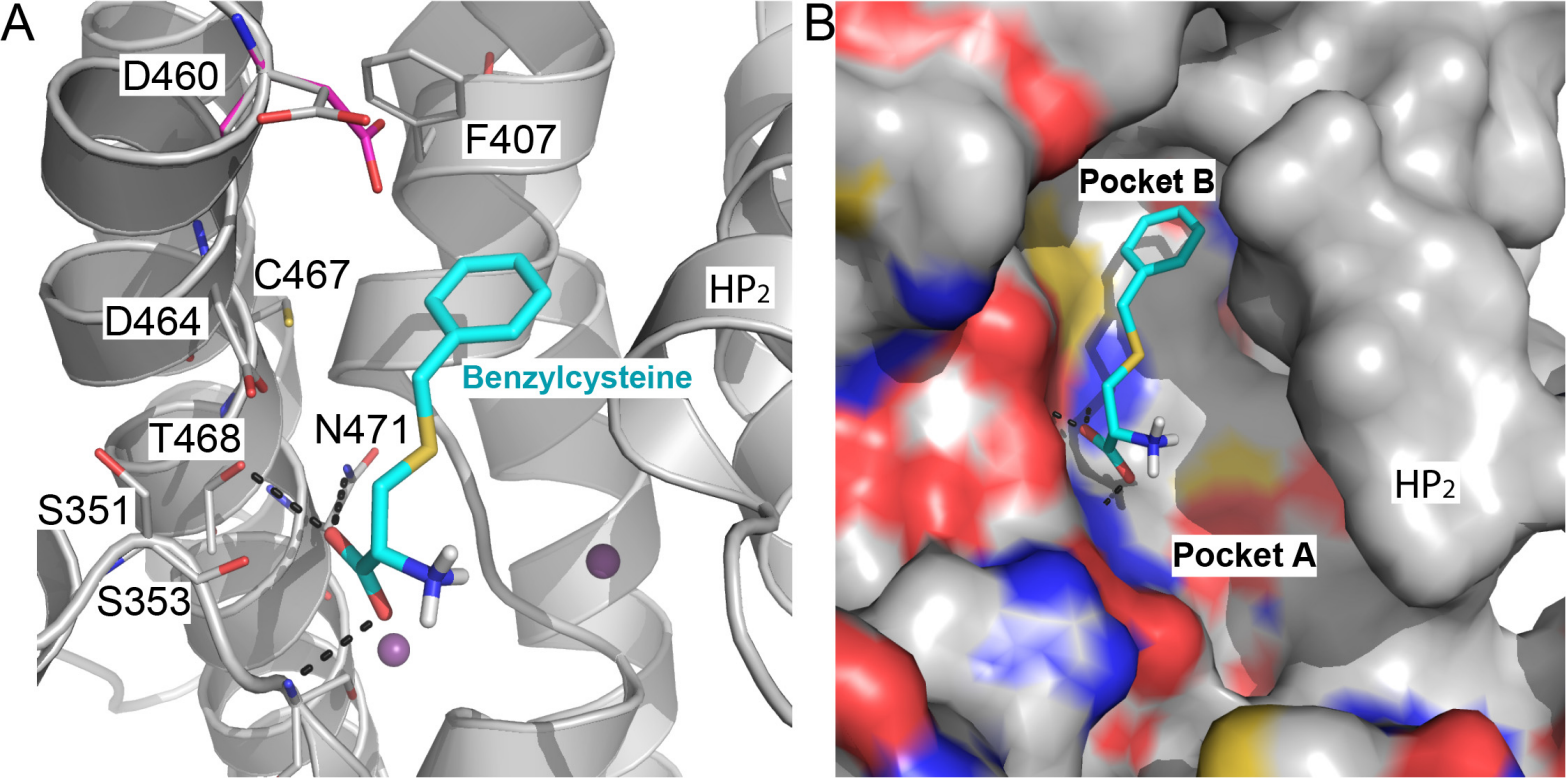
Outward-open conformation reveals an additional novel pocket. (**A**) Predicted binding mode of the known inhibitor benzylcysteine derived from docking. The backbone atoms of ASCT2 are visualized as gray cartoon, with key residues establishing hydrogen bonds with the ligand in gray sticks, with oxygen, nitrogen, and hydrogen atoms in red, blue, and white, respectively. Two potential rotamers of Asp460 including the orientation facing the binding site (in pink) and surface (gray) are shown as sticks. Sodium ions are represented in purple spheres. (**B**) The binding site surface of the outward-open model of ASCT2 bound to benzylcysteine reveals the novel pocket (pocket A) resulting from the opening of HP2.

The ASCT2 model in the outward-open conformation was based on Glt_Ph_ structure bound to the amino acid analog TBOA. TBOA is a weak inhibitor of Glt_Ph_ [11] but interacts with low micromolar affinities with mammalian glutamate transporters such as the EAATs. Thus, the model is thought to approximate a conformation that is incapable of transport. Our rationale was, therefore, that a model based on an inhibited conformation would be useful for identifying putative inhibitors. Some of the interactions between the ligands with the ASCT2 binding site are similar to the ASCT2-ligand interactions in the occluded model. In particular, the amino and carboxy groups of the amino acid ligands form polar interactions with the binding site residues Ser351 (backbone), Ser353, Asp464, Thr468, and Asn471 (Fig 6A). These interactions are also conserved with respect to the template structure, in which the amino acid analog TBOA interacts with the corresponding residues in Glt_Ph_ (i.e., Arg276, Arg278, Asp394, Thr398 and Asn401). Notably, two key features in the binding site of the outward-open conformation model likely contribute to its preference for amino acid-like ligands with a larger and more hydrophobic sidechain. First, HP2 is in an open conformation, leading to an increased surface accessible area in the ligand binding site compared to that of the occluded state (Fig 6B). The hydrophobic sidechain of TBOA occupies this additional binding pocket in the ASCT2 model (‘pocket A’), analogously to the Glt_Ph_ structure. Second, similarly to the occluded state model, Cys467 in ASCT2 replaces Arg397 in Glt_Ph_ to break a salt bridge with Asp460 (Asp390 in Glt_Ph_) and thus reveals the additional pocket accessible for binding (named ‘pocket B’ as in the occluded state) (Fig 6B). Therefore, we tested the hypothesis whether in the outward-open model, similarly to the occluded state model, Asp460 faces the binding pocket and directly interacts with the ligand (Fig 6A). However, logAUC scores for ASCT2 model with Asp460 sidechain facing the binding pocket and a model with this sidechain facing the surface were 27 and 11.1, respectively, indicating that the model with Asp460 sidechain facing the pocket cannot distinguish between known ASCT2 ligands and decoys any better than random. This suggests that, for this conformation, Asp460 is unlikely to be involved in ligand coordination and is more likely to face the surface (Fig 6A). Moreover, this result highlights the utility of iterative modeling and docking to refine structures, thereby capturing functionally important residues and regions on the protein surface.

Next, we docked various small molecule libraries from the ZINC database against the outward-open model. The binding site of this conformation is larger than that of the occluded model; therefore, the outward-open binding site is likely to accommodate inhibitors, which are larger than the molecules captured by the occluded model. We tested experimentally molecules that: i) interact with the conserved binding site residues that form the polar interactions with the amino and carboxy group of the known ligands (e.g., Ser351 and Asp464 interact with the amino moiety of the backbone of amino acids, whereas Ser353 and Asn471 interact with the carboxy moiety of the substrates); and molecules that ii) bind pockets A or B to increase the chemical space and binding affinity of potential hits. Overall three compounds matching those criteria were selected. Again, although we selected amino acid-like compounds, this transporter is highly specific, where molecules within one or two heavy atoms of known ligands do not necessarily interact with ASCT2 [14]. Moreover, these compounds are chemically dissimilar to known ASCT2 ligands, as measured by their Tanimoto Coefficient (Tc) values (Table 1 and S1 Fig). Furthermore, these putative ligands are predicted to bind newly identified pockets suggesting a novel mode of interaction of ASCT2 with small molecule ligands.

### Experimental characterization of predicted ligands suggests new inhibition mode

**Fig 7:**
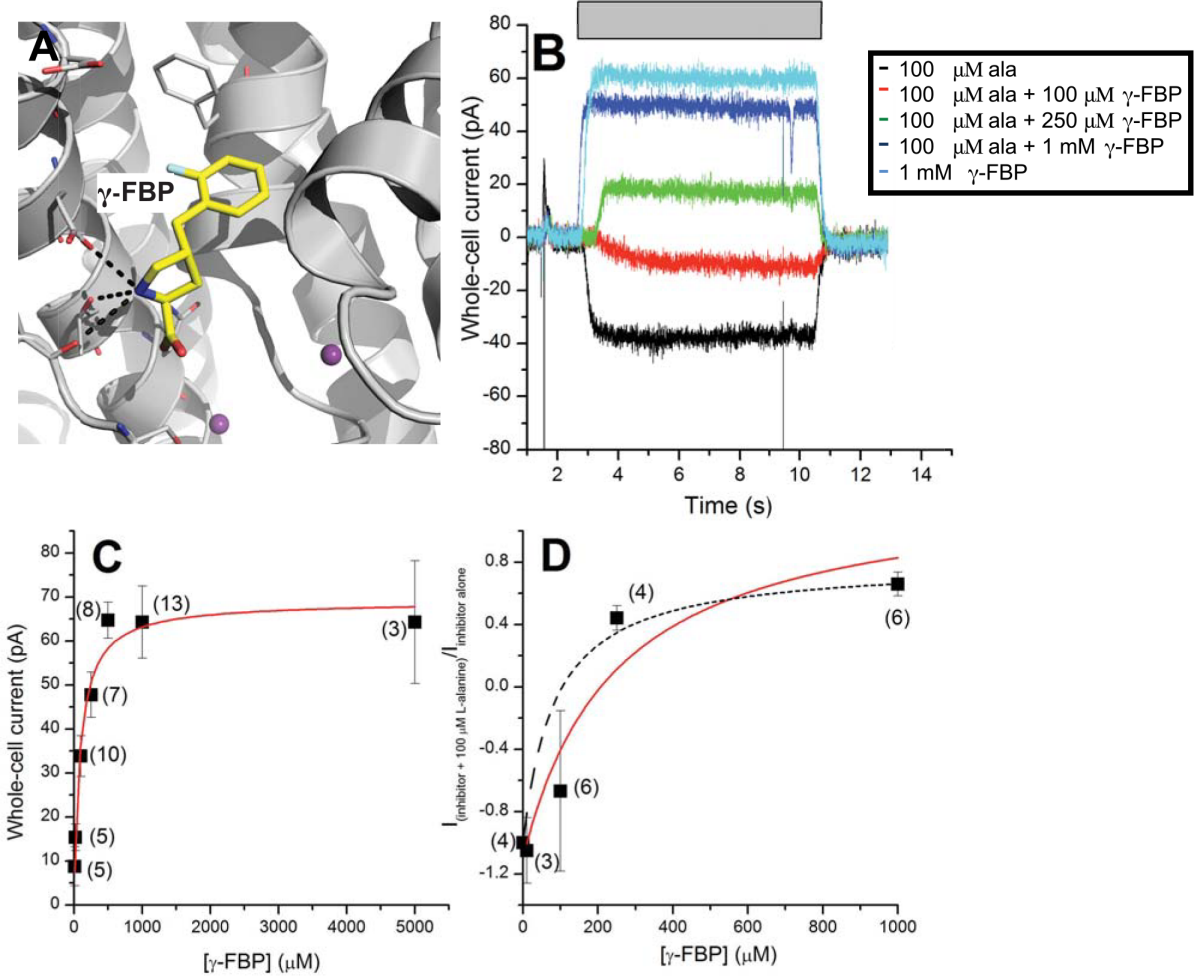
Identification of a potent ASCT2 inhibitor based on the outward-open model. (**A**) Predicted binding mode of the new identified inhibitor **γ**-FBP. The ASCT2 binding site is visualized as gray cartoon; sidechain atoms of key residues are illustrated with gray lines and the ligand is displayed as yellow sticks, with oxygen, nitrogen, and hydrogen atoms in red, blue, and white, respectively; hydrogen bonds between the inhibitor and ASCT2 (involving residues Ser351, Asp464, Thr468 and Asn471) are displayed as dotted black lines. (**B**) Representative current recordings in response to application of alanine, **γ**-FBP, and alanine + **γ**-FBP at conditions indicated in the legend. The gray bar depicts the duration of compound application. (**C**) Dose response relationship of **γ**-FBP-induced currents with the number of experiments averaged for each data point illustrated in brackets. (**D**) Alanine-induced currents (100 μM) in the presence of varying concentrations of **γ**-FBP (membrane potential = 0 mV, internal buffer contained 130 mM NaSCN and 10 mM alanine, external buffer contained 140 mM NaCl). The number of experiments averaged for each data point is illustrated in brackets. The dashed line represents the relationship based on a *K*_i_ of 87 μM, for comparison with (C).

Surprisingly, of the three selected compounds, the proline derivative **γ**-FBP (Fig 7A) was found to elicit large outward current (response of -0.64 ± 0.13 μM relative to saturating [alanine], Fig 7B and Table 1), indicating that it is an inhibitor of ASCT2 function. Consistent with this result, **γ**-FBP was able to alleviate alanine activation at concentrations down to 100 μM (Fig 7D). In addition, the apparent K_i_ for **γ**-FBP in the absence of alanine (87 ± 22 μM, Fig 7C and Table 1) was increased in the presence of alanine (Fig 7D, K_i_ of 250 ± 75 μM), an effect that is expected for competitive inhibition. If inhibition were purely non-competitive, the K_i_ would be expected to be independent of alanine concentration, in contrast to the experimental data.

**γ**-FBP is a chemically novel ligand for ASCT2, exhibiting Tc of 0.47 to the most closely related known ASCT2 ligand (Table 1). The result that a proline derivative is the most potent inhibitor discovered in this study is particularly intriguing because proline is not an ASCT2 ligand (Fig 5E) and because a different proline derivative was shown to activate ASCT2 (Fig 5E and Table 1). This unexpected activity of **γ**-FBP can be explained by a novel hydrophobic scaffold that includes an aromatic ring capable of forming π-π interactions with Phe407 of pocket B, and a fluorine atom (Fig 7A). Fluorine atoms have been shown to improve the therapeutic activities of drugs by enhancing their affinities in various ways, one of them being the interaction of the fluorine with the peptide bond [40]. In this particular case, the fluorine atom points toward the Val364-Asp365 peptide bond (Fig 7A). In summary, the outward-open model has a larger binding site facilitating targeting additional pockets for drug discovery. Future studies are expected to optimize the binding of this new scaffold to design more potent inhibitors of ASCT2.

### Effects of ligands on ASCT2 mediated glutamine uptake and cell viability in C8161 melanoma cells

**Fig 8:**
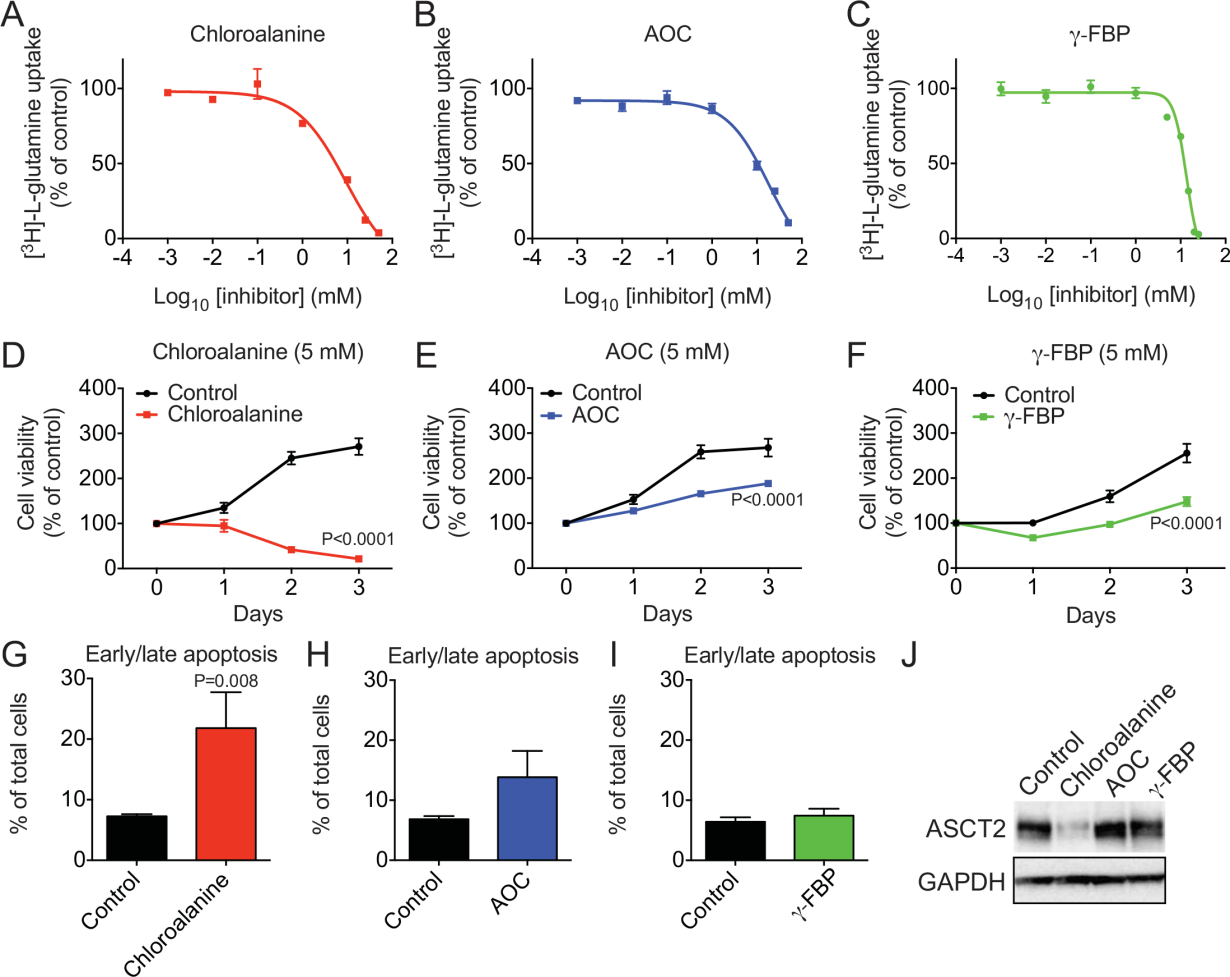
Chloroalanine, AOC and γ-FBP inhibit ASCT2-mediated glutamine uptake and cell viability in C8161 human melanoma cells. (**A**-**C**) [^3^H]-L-glutamine uptake in C8161 cells was used to determine the IC_50_ of chloroalanine, AOC and **γ**-FBP (n = 3). (**D**-**F**) MTT cell viability assay (n = 3) in C8161 cells incubated with chloroalanine (5 mM), AOC (5 mM) and **γ**-FBP (5 mM). Two-way ANOVA test was performed to determine significance. (**G**-**I**) Apoptosis (Early, Annexin V+ PI-; Late, Annexin V+PI+) was examined by flow cytometry in C8161 cells incubated with chloroalanine (5 mM), AOC (5 mM) and **γ**-FBP (5 mM). Mann Whitney U test was used to determine significance. (J) ASCT2 expression (with GAPDH as a loading control) in C8161 cells was assessed after 48 hours incubation with chloroalanine (5 mM), AOC (5 mM) and **γ**-FBP (5 mM).

We have previously shown that ASCT2 mediates glutamine uptake in C8161 human melanoma cells, and that blocking ASCT2 activity inhibits cell growth and viability (Methods) [6]. We therefore used this model to confirm the activity of three ASCT2 ligands: Chloroalanine, and aminooxetane-3-carboxylate (AOC), two of the most effective activators, and **γ**-FBP, the most potent inhibitor ligand for ASCT2 inhibition (Fig 5E). In the presence these compounds, [^3^H]-L-glutamine uptake was decreased in a dose-dependent manner in C8161 cells, in agreement with their elicitation of current as activators and potential substrates (Fig 8A–8C). The IC_50_ of chloroalanine was 9.2 mM, and the IC_50_ of AOC and **γ**-FBP was between 10–15 mM, which is similar to the ASCT2 ligand benzylserine (IC_50_ of 5.3 mM in C8161 cells) [6].

Glutamine is a conditionally essential amino acid required for cancer cell growth, being used as fuel source for the TCA cycle, and a carbon and nitrogen source for macromolecule production. We therefore examined cell viability in the presence of chloroalanine (5 mM), AOC (5 mM) and **γ**-FBP (5 mM) using an MTT assay in C8161 cells (Methods). Chloroalanine, AOC and **γ**-FBP significantly inhibited cell viability in C8161 cells (Fig 8D–8F). To determine whether these compounds also induced apoptosis, we used flow cytometry to quantitate Annexin V/propidium iodide staining in C8161 cells exposed to chloroalanine (5 mM), AOC (5 mM) and **γ**-FBP (5 mM) for 48 hours (Fig 8G–8I). While chloroalanine significantly increased apoptosis, neither AOC nor **γ**-FBP led to a significant increase in early (Annexin V+PI-) or late (Annexin V+PI+) apoptosis. Inhibition of intracellular amino acid levels has been shown to induce adaptive responses through ATF4 transcriptional regulation of amino acid transporters including ASCT2 [3,8].

To ensure these inhibitory effects were not due to changes in ASCT2 protein levels, we examined ASCT2 protein levels by Western blotting 48 hours after incubation with chloroalanine (5 mM), AOC (5 mM) or **γ**-FBP (5 mM) (Fig 8J). While chloroalanine reduced the levels of ASCT2 protein, there was no change after AOC or **γ**-FBP, suggesting the inhibitory effects are directly on ASCT2 transport rather than protein expression. The low levels of ASCT2 after chloroalanine are likely due to apoptosis, which was significantly increased in chloroalanine treated cells, but not AOC or **γ**-FBP treated cells. Although these previously unknown ASCT2 ligands exhibit weaker therapeutic effect on the melanoma cell line and are thus unlikely to be used for drug development, these compounds can be useful chemical tools to further characterize the role of ASCT2 in cancer metabolism. For example, **γ**-FBP is an inhibitor that deprives the cancer cells from nutrients and the activator Chloroalanine can potentially be further developed as a cytotoxic ligand.

### Conclusions

ASCT2 is a sodium-dependent neutral amino acid exchanger located in peripheral tissues, which is highly expressed in a variety of cancers where it provides key nutrients and signaling molecules for growth and proliferation. ASCT2 can be a drug target for inhibitors that block nutrient uptake or it can import cytotoxic substrates to act on a different target. Here, we describe the structural models of ASCT2 in two distinct conformations and identify specificity determinants for this protein. Three major findings emerge from this study.

First, we identified seven previously unknown ASCT2 ligands, including five activators and two inhibitors. This result provides chemical basis for discriminating inhibitors from activators as well as novel scaffolds for optimizing more efficacious ligands against ASCT2, an emerging drug target for cancer and neurological disorders. Moreover, three of these compounds were chemically different from known ligands (acivicin) or chemically related to known non-ligands (Table 1). For example, the activator *cis*-3-hydroxyproline and inhibitor **γ**-FBP are proline derivatives, and we confirmed that proline is not an ASCT2 ligand (Fig 5). For the activator, the hydroxyl group C_β_ of the proline enables additional interaction with residue Asn471, but the molecule remains small enough to activate the transporter and possibly get transported (Fig 4D). Conversely, for the inhibitor, a fluorobenzyl is bound to the C_γ_ of the proline (Fig 7A). This bulky group is likely to enhance the affinity of the molecule through π-π and fluorine-peptide bond interactions, but the molecule is too large to be transported. Notably, structure based virtual screening enabled us to identify seemingly non-ligands.

Second, three of our hits (choloroalanine, AOC and **γ**-FBP) inhibited glutamine uptake and proliferation of the melanoma cell line C8161, at similar concentrations to the ASCT2 inhibitor benzylserine (Fig 8). Interestingly, the cytotoxic compound acivicin, which also interacts with the structurally unrelated cancer-associated amino acid transporter LAT-1 was previously shown to inhibit proliferation of GBM cell line [22]. Acivicin is not a strong inhibitor of any of these transporters, however, it likely obtains its positive and negative pharmacological effects by acting on more than one target via polypharmacology. Indeed, acivicin was recently shown to interact with additional metabolic enzymes suggesting that polypharmacology contributes to its therapeutic effects [41,42]. Thus, future drugs can potentially be developed by refining their interaction with multiple transporters and other targets simultaneously.

Third, we used iterative modeling and docking approach to model ASCT2 structure based on X-ray structures of a prokaryotic aspartate transporter that shares about 24% sequence identity with ASCT2 and different substrate specificity. This analysis of a technically challenging target provides a framework for understanding amino acid selectivity among the SLC1 family of amino acid transporters that play an important role in various biological activities such as neurosignaling, as well as an approach that is generally applicable to the characterization of other human transporter structures and their interactions with ligands, including drugs.

Finally, many solute carriers (SLC) play a key role in various human diseases by mediating transport of amino acids, sugars (e.g., GLUTs), and other metabolites such as citric-acid cycle intermediates [12]. Such disorders result from dysregulated metabolism that can be caused by single point mutations or aberrant expression levels. Therefore, nutrient SLC transporters are emerging drug targets for both targeting and delivery. For instance, the Food and Drug Administration has recently approved the type-2 diabetes drug canagliflozin, which acts by inhibiting transport by Na^+^-glucose co-transporter 2 (SGLT2/SLC5A2) to lower concentration of sugar in the blood [43]. The significant increase in the number of membrane transporter structures, coupled with the progress computer-aided drug design methodologies, has improved the applicability of rational structure-based drug-design to human SLCs [9]. Importantly, even though human structures are determined there is still need to characterize computationally and experimentally additional conformations of transporters so additional fractions of the chemical space with virtual screening can be covered, as demonstrated in this study. Taken together, in the next few years, the number of drugs targeting SLC transporters that are designed rationally with combined computational and experimental techniques is expected to grow significantly.

## Materials and Methods

### Homology modeling

We modeled ASCT2 based on X-ray structures of Glt_Ph_ from *Pyroccocus horikoshii* in the occluded and the outward-open conformations (PDB identifiers 2NWX and 2NWW, respectively). The initial alignment between ASCT2 and Glt_Ph_ was calculated using the Promals3D server using various parameters [44] and then subsequently refined based on comparison to previously published comprehensive alignment of SLC1 members with Glt_Ph_ [10], as well as by constructing models based on the various alignments and analyzing them visually [45]. Three long and divergent segments, including the N-terminus (53 residues), the loop between transmembrane helix (TM) 3 and TM4a (29 residues), the loop between TM4b and TM4c (27 residues), and the C-terminus residues (55 residues) were not included in our model [10]. These regions are distant from the binding site and are unlikely to interact with the ligand. 385 and 387 residues of ASCT2 were modeled for the occluded and outward-open models respectively, covering 70% and 71% of the protein sequence.

For each conformation, we used the standard ‘automodel’ class of MODELLER-9v11 [25] to generate initial 100 models, which were evaluated using Z-DOPE, a normalized atomic distance-dependent statistical potential based on known protein structures [46]. The Z-DOPE score of the top models were -0.19 for the occluded model and -0.08 for the outward-open model, suggesting that the models are likely to be sufficiently accurate to guide further structure/function studies [9,46,47]. Moreover, the models were constructed with non-protein atoms, based on their coordinates in the template structures. These non-protein elements include the sodium ions, as well as the ligand molecules, aspartate and TBOA for the occluded and outward-open models, respectively. Finally, we sampled additional conformations for enrichment calculations by sidechain modeling and energy minimization with MD simulations.

### Molecular Dynamics (MD) simulations

MD simulations were performed with GROMACS4 [27]. Each model was refined using the following protocol. The model was subjected to 10,000 steps of conjugate gradient minimization using the Amber99SB-ILDN force field [48,49]. To account for the membrane hydrophobic environment, an implicit model for the solvent based on a generalized Born formalism was used. A dielectric constant equal to 2 was used to model the membrane interior. Whenever ions were present in the model, the system was simulated “in vacuum” and the coulomb interactions were screened by using a dielectric constant equal to 2. All bond lengths were constrained to their equilibrium values using the LINCS algorithm [50] and a time step of 2 fs was adopted. A cutoff of 1.0 nm was used for the Lennard-Jones and the electrostatic interactions.

### Ligand docking and enrichment

Initial docking and enrichment calculations were performed with DOCK, as described previously [51–53]. The homology models were evaluated by calculating the AUC (Area Under the Curve) and LogAUC of the enrichment plots representing the ability of the virtual screening to discriminate known ligands among the set of decoys. The plots show the percentage of known ligands correctly predicted (y-axis) within the top ranked subset of all database compounds (*x*-axis on logarithmic scale) (Fig 2) [28,29,54]. 28 known ligands of ASCT2 were selected from the literature [14,55,56] and 1,400 decoys were generated with the DUD-E server [57]. This set of 1,428 molecules was then screened against three models with OpenEye FRED[58]: i) the occluded conformation model ii) the outward open conformation, iii) the outward open conformation with Asp460 facing the binding site.

### Docking with FRED

OpenEye FRED uses an empirical- and shape-based scoring function [58]. The receptor was prepared with the MAKE_RECEPTOR utility of FRED. The box enclosing the binding site was generated based on the coordinates of the crystallographic ligand (aspartate and TBOA for the occluded outward-open conformations, respectively). The docked poses were ranked by the Chemgauss4 scoring function, which is defined by smoothed Gaussian potentials describing the complementarity (by shape and chemical properties) between the ligands and the binding site.

### Ligand libraries for virtual screening

We used DOCK and FRED to virtually screen the following compound libraries from ZINC: (i) A filtered version of the Kyoto Encyclopedia of Genes and Genomes (KEGG) DRUG database that included 6,436 approved drugs in Japan, USA, and Europe, as well as over the counter (OTC) drugs [52]. (ii) A filtered version of the KEGG LIGAND COMPOUND database that included 12,730 metabolites, biopolymers, and other bioactive molecules. For example, molecules containing 50 or more nonhydrogen atoms or a molecular weight greater than 600 Da were filtered out, because docking does not typically work well for such large molecules. (iii) The ZINC Fragment Now set that included 575,000 compounds with molecular weight (mwt) of 250 Dalton or lower, five or fewer rotatable bonds (rot), and xlogP value of 3.5 or lower, where xlogP is the octanol/water partition coefficient (logP) calculated by an atom additive method. iv) The ZINC Leads Now set that contained 2,268,809 compounds of molecular weights between 250 and 350 Dalton, 7 or fewer rotatable bonds and xlopP value of 3.5 or lower [29,59].

### HEK293 culture and transfection for electrophysiology

cDNA coding for rat ASCT2 was kindly provided by S. Bröer [60]. The coding region of the cDNA was subcloned into the EcoR*I* site of the pBK-CMV vector (Stratagene). The cDNA construct was used to transiently transfect human embryonic kidney cells (HEK293, ATCC No. CGL 1573) with leptrime reagent (Polyplus, Ilkrich, France). Transfection was performed as detailed in the supplier’s instructions. Electrophysiological recordings were performed 24–48 h after transfection.

### Electrophysiological methods

ASCT2-associated whole-cell currents were recorded with an Adams & List EPC7 amplifier (HEKA, Lambrecht, Germany) using voltage-clamp conditions. Open-tip electrode resistances were 2–3 MΩ and the series resistance (*R*_S_) was 5–8 MΩ. *R*_S_ was not compensated in the recordings, as compensation had no effect on the magnitude of the observed currents. The extracellular bath solution contained (in mM): 140 NaMES, 2 MgGluconate_2_, 2 CaGluconate_2_, and 10 HEPES (MES = methanesulfonic acid, pH 7.4 / NaOH). The pipette solution contained (in mM): 130 NaSCN, 2 MgCl_2_, 10 EGTA, 10 HEPES, and 10 L-alanine/cysteine (pH 7.3 / NaOH). Using this intracellular solution, the transporters operate in the Na^+^/alanine exchange mode. Here, external alanine is exchanged with internal alanine in the absence of net transport. The exchange mode is associated with the activation of an uncoupled anion current, which was used as an indirect measure of transport activity [14,36]. The currents were low pass filtered at 3 kHz (EPC7 built-in filter) and digitized with a digitizer board (Digidata 1200, Axon Instruments, Foster City, CA, USA) at a sampling rate of 10–50 kHz (controlled by software, Axon PClamp7). All experiments were performed at room temperature. Rapid solution exchange was essentially performed as described previously [61]. Briefly, substrates and inhibitors were applied to the ASCT2-expressing HEK293 cells with a quartz tube (350 μm tube diameter) positioned at a distance of ≈0.5 mm to the cell. The linear flow rate of the solutions emerging from the opening of the tube was approximately 5–10 cm/s.

### Melanoma cell culture for uptake, proliferation and apoptosis studies

Human melanoma cell line C8161 culture was performed as previously described [6]. Media used was DMEM/F12 medium (Life Technologies) containing 5% (v/v) fetal bovine serum (FBS), penicillin-streptomycin solution (Sigma-Aldrich). Cells were maintained at 37°C in a fully humidified atmosphere containing 5% CO_2_. Inhibitors (chloroalanine, AOC and **γ**-FBP) were resuspended in H_2_O, with control cells treated with the appropriate concentrations of vehicle alone.

### Glutamine uptake assay

C8161 cells (1 × 10^5^/well) were incubated with [^3^H]-L-glutamine (400 nM; PerkinElmer) in MEM media (Life Technologies) for 15 min at 37°C in the presence or absence of each inhibitor. Cells were collected and transferred to filter paper using a 96-well plate harvester (Wallac PerkinElmer), dried, exposed to scintillation fluid and counts measured using a liquid scintillation counter (PerkinElmer).

### Cell viability assay

MTT (3–(4,5–dimethylthiazol–2–yl)–2,5–diphenyl tetrasodium bromide; Millipore) cell viability assay was performed as previously described [62]. Briefly, C8161 cells (1 × 10^4^/well) were seeded in a flat-bottomed 96–well plate, incubated overnight in media, prior to culture with or without each inhibitor. MTT solution (10 μL) was added to each well for 4 h, prior to addition of 100 μL of isopropanol/HCl solution. Solution was mixed thoroughly and plates immediately read at 570 nm/630 nm in a PolarStar plate reader (BMG). Results were plotted as percentages of the absorbance observed in control wells.

### Annexin V assay

Cells (5 × 10^5^ per well) were seeded in 24 well plates, allowed to adhere overnight, and then incubated in the presence of 5 mM chloroalanine, AOC or **γ**-FBP for 48 hours. Cells were detached using Tryple and resuspended in 300 μL of binding buffer (HEPES-buffered PBS supplemented with 2.5 mM calcium chloride) with Annexin V-APC (BD) and incubated for 15 min in the dark at room temperature. Propidium iodide solution (5 μg/mL) was added, and the cells were analyzed using the BD LSRFortessa and FlowJo software.

### Western blotting

Cells (5 × 10^5^ per well) were seeded in 24 well plates, allowed to adhere overnight, and then incubated in the presence of 5 mM chloroalanine, AOC or **γ**-FBP for 48 hours. Cells were detached using Tryple and lysed in lysis buffer (50 μL; 20 mM Tris-HCl, 150 mM NaCl, 1% Triton X-100, 0.5% Na deoxycholate, 0.1% SDS) with protease inhibitor Cocktail III (Bioprocessing Biochemical, California). Protein was measured using micro-BCA method (Pierce) and loaded onto a 4–12% gel (Life Technologies), electrophoresed, and transferred to PVDF membrane. The membrane was blocked with 2.5% (w/v) BSA in PBS-Tween20, and incubated with primary (ASCT2, Cell Signaling; GAPDH, Abcam) and secondary antibodies. Secondary HRP-labeled antibodies (Millipore) were detected using chemiluminescence reagents (Pierce) on a Kodak imager (Kodak).

## Supporting Information

S1 Fig 2D representation of the experimentally confirmed ligands. (PDF)

S2 Fig Exchange current is mediated by a transported substrate, but not by a non-transported inhibitor. Voltage jumps induce transport-mediated exchange current (due to voltage-dependent re-equilibration of the translocation equilibrium) in the presence of the transported substrate alanine (1 mM, black trace), but not in the presence of the non-transported inhibitor benzylserine (5 mM, red trace). The solutions contained 140 mM Na^+^ and 10 mM alanine (intracellular) and 140 mM Na^+^ (extracellular). The anion was methanesulfonate, which does not permeate the anion conductance.
(PDF)

S1 Models PDB files of the ASCT2 models in occluded (cm251) and outward-open (cm301) conformations. (ZIP)
