# Supplementary material for: Ligand Discovery for the Alanine-Serine-Cysteine Transporter (ASCT2, SLC1A5) from Homology Modeling and Virtual Screening
Source: PLoS Comput Biol. 2015 Oct 7;11(10):e1004477. doi: 10.1371/journal.pcbi.1004477 (PMC4596572; doi:10.1371/journal.pcbi.1004477)
Supplement: S1 Fig — (PDF) [file pcbi.1004477.s001.pdf]

Name	Sketch
Alanine (control)	
cis-3-hydroxyproline	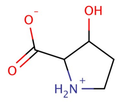
Aminooxetanecarboxylate (AOC)	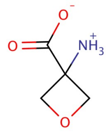
Penicillamine	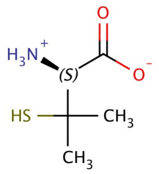
Chloroalanine	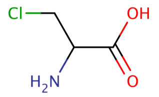
Acivicin	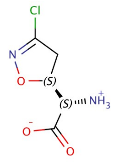
L-DOPS	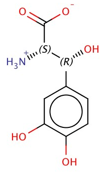
γ-FBP	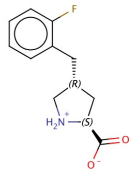

S1 Fig. 2D representation of the experimentally confirmed ligands
