# Supplementary material for: Ligand Discovery for the Alanine-Serine-Cysteine Transporter (ASCT2, SLC1A5) from Homology Modeling and Virtual Screening
Source: PLoS Comput Biol. 2015 Oct 7;11(10):e1004477. doi: 10.1371/journal.pcbi.1004477 (PMC4596572; doi:10.1371/journal.pcbi.1004477)
Supplement: S2 Fig — Voltage jumps induce transport-mediated exchange current (due to voltage-dependent re-equilibration of the translocation equilibrium) in the presence of the transported substrate alanine (1 mM, black trace), but not in the presence of the non-transported inhibitor benzylserine (5 mM, red trace). The solutions contained 140 mM Na+ and 10 mM alanine (intracellular) and 140 mM Na+ (extracellular). The anion was methanesulfonate, which does not permeate the anion conductance. (PDF) [file pcbi.1004477.s002.pdf]

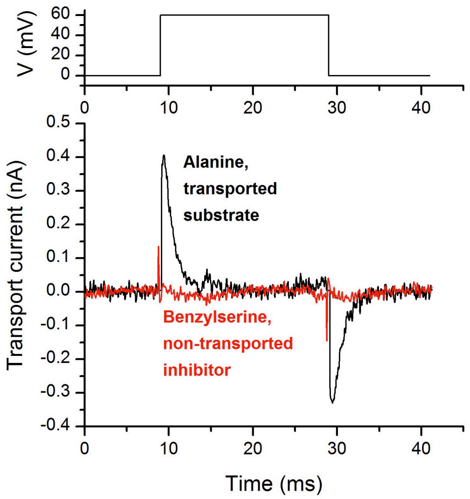

S2 Fig. Exchange current is mediated by a transported substrate, but not by a non-transported inhibitor.

Voltage jumps induce transport-mediated exchange current (due to voltage-dependent re-equilibration of the translocation equilibrium) in the presence of the transported substrate alanine (1 mM, black trace), but not in the presence of the non-transported inhibitor benzylserine (5 mM, red trace). The solutions contained 140 mM Na^+ and 10 mM alanine (intracellular) and 140 mM Na^+ (extracellular). The anion was methanesulfonate, which does not permeate the anion conductance.
